# Integrated analysis of the functions and prognostic values of RNA binding proteins in hepatocellular carcinoma

**DOI:** 10.1186/s12876-021-01843-0

**Published:** 2021-06-15

**Authors:** Zeng-Hong Wu, Hong-Ming Huang, Dong-Liang Yang

**Affiliations:** Department of Infectious Diseases, Union Hospital, Tongji Medical College, Huazhong University of Science and Technology, Wuhan, 430022 China

**Keywords:** Hepatocellular carcinoma, RNA binding proteins, TCGA, Prognosis

## Abstract

**Background:**

Hepatocellular carcinoma (HCC), one of the most common malignant tumors worldwide, ranks as the fifth most common cancer and has been the second most frequent cause of cancer-related death. RNA binding proteins (RBPs) are proteins that interact with different classes of RNA and are commonly detected in cells.

**Methods:**

We used RNA sequencing data from TCGA to display dysfunctional RBPs microenvironments and provide potential useful biomarkers for HCC diagnosis and prognosis.

**Results:**

330 differently expressed RBPs (208 upregulated and 122 downregulated) were identified. KEGG were mainly enriched in RNA degradation, Influenza A, Hepatitis C, RIG-I-like receptor signaling pathway, Herpes simplex virus 1 infection and RNA transport. CBioPortal results demonstrated that these genes were altered in 50 samples out of 357 HCC patients (14%) and the amplification of *BRCA1* was the largest frequent copy-number alteration.

**Conclusion:**

Based on the online database, we identified novel RBPs markers for the prognosis of hepatocellular carcinoma.

**Supplementary Information:**

The online version contains supplementary material available at 10.1186/s12876-021-01843-0.

## Background

Hepatocellular carcinoma (HCC), one of the most common malignant tumors worldwide, ranks as the fifth most common cancer and has been the second most frequent cause of cancer-related death with over 500,000 newly diagnosed per year [1]. Nonalcoholic steatohepatitis and viral hepatitis are the most common causes of cirrhosis, with approximately four fifths of cases progressing to liver cancer [2, 3]. Owing to HCC recurrence, the prognosis for HCC is also dismal, and the 5-year overall survival (OS) rate is below 50% [4]. Notwithstanding rapid advances in medical technology, there is still no effective treatment strategy for HCC patients [5]. Serum markers such as alpha-fetoprotein (AFP) are known to be used in clinical practice, but they are not adequate enough in specificity and sensitivity [6]. Therefore, it is necessary to search effective biomarkers for the diagnosis and treatment of HCC patients.

RNA-binding proteins (RBPs) are proteins that interact with various kinds of RNA and are commonly detected in cells. RBP affects post-transcription and regulates cell physiology, and is involved in controlling RNA stability, alternative splicing, translation, apoptosis, modification, and localization [7]. A total of 1542 RBPs were identified in human cells by high-throughput screening, accounting for 7.5% of all protein-coding genes [8]. Over the past few decades, genome-wide techniques have shown that many RBPs functions are abnormal in cancers and their expressions are associated with disease prognosis. Kudinov et al. [9] reported that RBP Musashi-1 (MS1) and Musashi-2 (MS2) can operate necessarily in oncogenic signaling pathways, involving in NUMB/Notch, TGFβ/SMAD3, PTEN/mTOR, cMET, MYC, etc. Similarly, study revealed *RPS3* in the post-transcriptional can regulate the expression of *SIRT1* to promote hepatocarcinogenesis [10]. Study also demonstrated that RBPs level was an independent risk factor involved in early intrahepatic recurrence of HCC among 2 years after surgery and suppression of RBPs obviously suppressed cell invasion as well as proliferation [11]. However, only a small number of RBPs have been studied in depth, and so far, RBPs have been found to play a key role in cancer. At the same time, there is no features to systematically assess cancer-related RBPs and predict OS in HCC patients. Therefore, we used RNA sequencing (RNA-seq) data based on the Cancer Genome Atlas (TCGA) to reveal abnormalities in the microenvironment of RBPs, the potential molecular function, the clinical significance of RBPs, and to furnish prospective beneficial biomarkers for HCC prognosis.

## Methods

### Data gathering

RNA-seq data from 424 (50 normal and 374 tumor) and comparative clinical data were identified and downloaded from the level 3 gene expression information (standardized FPKM) of the TCGA-HCC cohort. The collected clinical pathological data included gender, age, stage, grade, TMN classification, survival status and number of days of survival. 1542 RBPs were get from Gerstberger et al. [7] study, the detailed information was shown in Additional file 2: Table S1. HCC patients (231 patients) from the ICGC (LIRI-JP) cohort were used for validation.

### Building and validating the prognostic RBPs-based signature

After normalized the mRNA expression profiles through edgerR (R package). |log_2_FC|≥ 0.5 and FDR < 0.05 were determined as differently expressed RBPs. Through caret (R package) with function create data partition divided HCC cohort into two groups: a training set and a testing set. A univariate COX regression model was built via the RBPs’s levels, T, N, M, age, gender, stage, and survival information in the training cohort. Then, using significant RBPs got from univariate COX regression models and clinical factors to multivariate COX proportional hazard regression models. At the same time, RBPs’s levels that were significant both in univariate and multivariate Cox regression analysis were choose as characteristically RBPs. The prognostic signature as risk score = (Coefficient RBP1 × expression of RBP1) + (Coefficientm RBP2 × expression of RBP2) + ⋯ + (Coefficient RBPn × expression RBPn). Receiver operating characteristic (ROC) curve was plotted to prediction accuracy of the prognostic signatures for HCC patients.

### Enrichment analysis of prognostic RBPs

Cytoscape is used to visualize the interaction network between protein–protein interaction (PPI). The plug-in Molecular Complex Detection (MCODE) of Cytoscape was applied to selected the most significant module in the PPI networks. The Kyoto Encyclopedia of Genes and Genomes (KEGG) is a database for exploring high-level gene functions and associating genomic data from large-scale molecular datasets. Gene ontology (GO) function analysis (biological processes (BP), cellular components (CC) and molecular functions (MF) is a tool to analyze biological process and annotate genes. We explored the function of the identified both upregulated and downregulated RBPs biological analyses using GO and KEGG via R language ggplot2 package.

### Externally validated prognostic RBPs signatures

Tumor IMmune Estimation Resource (TIMER) database used to compared mRNA level between different cancers; The Human Protein Atlas database (HPA) further proved the expression of prognostic genes and CBioportal to study genetic alterations. All data processing based on *P* less than 0.05.

### Statistical analysis

Statistical analyses were performed using R (version 3.5.3) and R bioconductor software packages. Benjamini–Hochberg's method was used to convert *P* values to FDR. Perl language was used for data matrix and data processing and a *P* value less than 0.05 was used. Normally and non-normally distributed variables were analyzed using the unpaired student’s *t* test and the Wilcoxon test, respectively. The relationship between our model and clinicopathological manifestations was evaluated using chi-square test.

## Results

### Enrichment analysis of identified RBPs

**Fig. 1:**
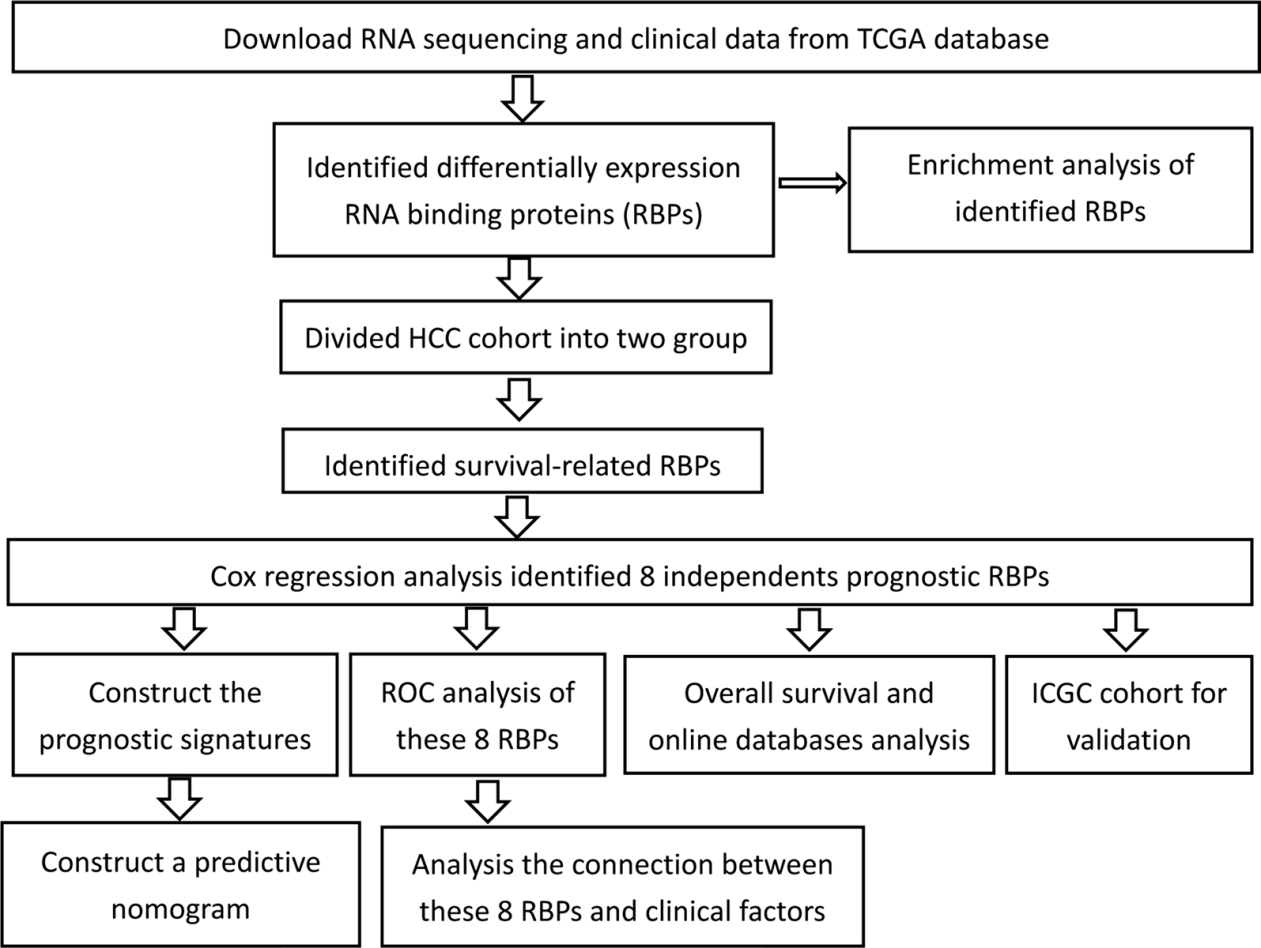
The flow chart of this study scheme

**Fig. 2:**
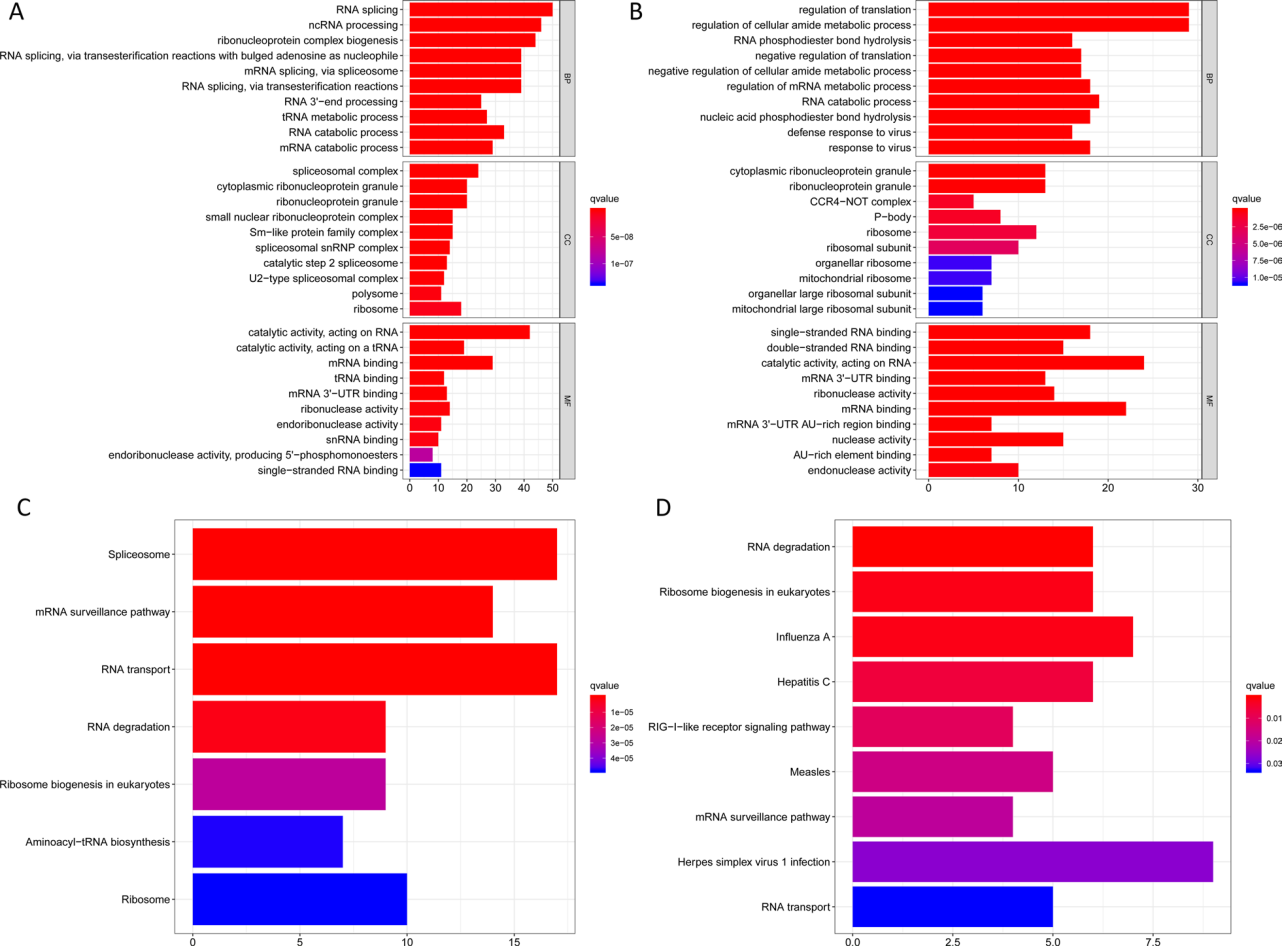
The function of the identified upregulated and downregulated RNA binding proteins, respectively. **A** GO results of upregulated RBPs; **B** KEGG results of upregulated RBPs; **C** GO results of downregulated RBPs; **D** KEGG results of downregulated RBPs

Figure 1 presents a flow chart of this study scheme. We first identified 330 differently expressed RBPs (208 upregulated and 122 downregulated Additional file 2: Table S2). We next explored the function of the identified upregulated and downregulated RBPs, respectively. In upregulated RBPs group, BP of target genes were enriched in RNA splicing, ncRNA processing, tRNA metabolic process, RNA localization, tRNA processing; MF were mainly enriched in catalytic activity, mRNA/tRNA/ snRNA binding, ribonuclease activity; CC were mainly enriched in spliceosome complex, cytoplasmic ribonucleoprotein granule, ribonucleoprotein granule; KEGG were mainly enriched in spliceosome, mRNA surveillance pathway, RNA transport, RNA degradation, Ribosome. In addition, in downregulated RBPs group, BP of target genes were enriched in regulation of translation, regulation of cellular amide metabolic process, RNA phosphodiester bond hydrolysis; MF were mainly enriched in single-stranded RNA binding, double-stranded RNA binding, catalytic activity, acting on RNA, mRNA 3'-UTR binding; CC were mainly enriched in cytoplasmic ribonucleoprotein granule, ribonucleoprotein granule, CCR4-NOT complex, P-body, ribosome; KEGG were mainly enriched in RNA degradation, Influenza A, Hepatitis C, RIG-I-like receptor signaling pathway, Herpes simplex virus 1 infection, RNA transport Fig. 2 and Additional file 2: Table S3.

### PPI network construction and hub module screening

**Fig. 3:**
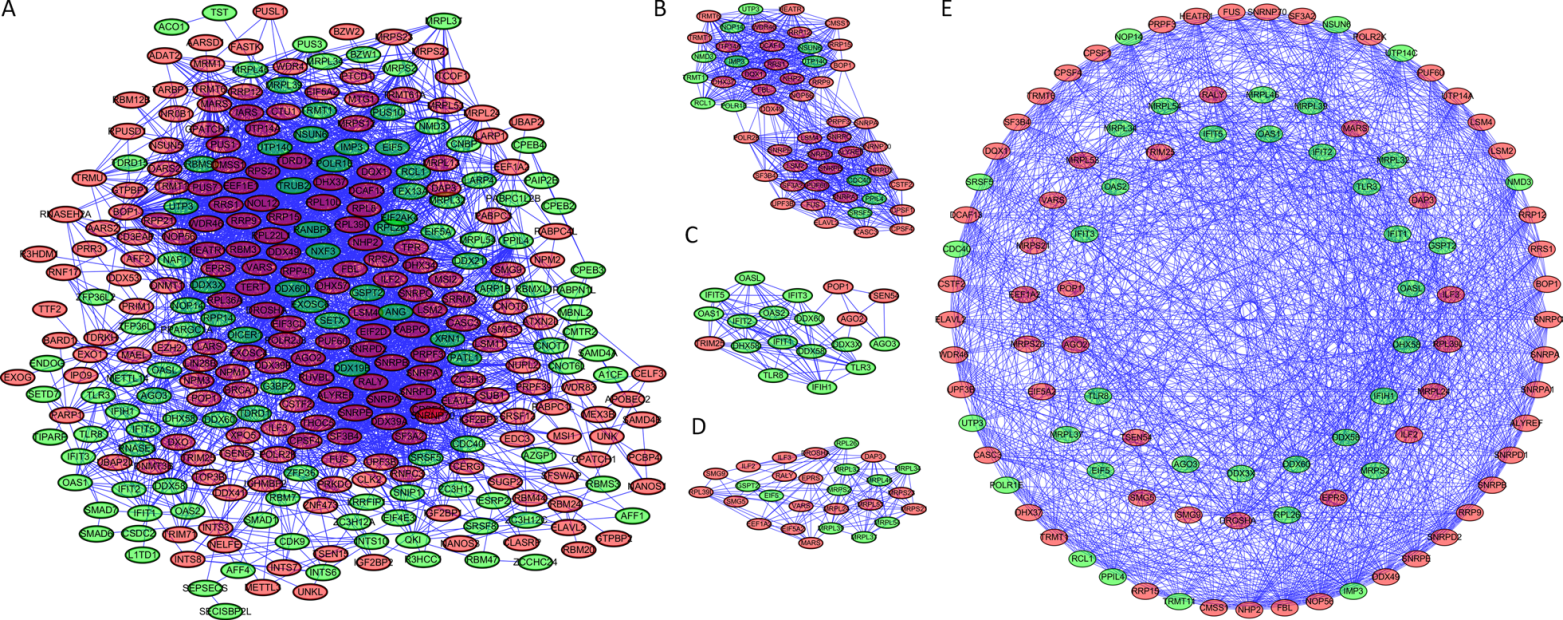
Protein–protein interaction (PPI) network construction and key module screening. **A** PPI network; **B**–**D** key module; **E** PPI network construction using all the genes from 3 key modules

To better known the possible molecular functions of these differentially expressed RBPs, we constructed the PPI using Cytoscape the STRING database, and via MCODE to selected hub genes. Finally, 11 hub genes (*BLAVL2, IFIT5, EEF1A2, RFR21, CDX9, PABPC1L, TEX13A, NUPL2, DARS2, RNF17,* and *PARP1*) and top 3 significant modules. The PPI network contained overall 311 nodes and 2942 edges (Fig. 3A); module 1 involved in 53 nodes and 709 edges (Fig. 3B); module 2 included 19 nodes and 97 edges (Fig. 3C); and module 3 included 27 nodes and 134 edges (Fig. 3D). We next extracted and combined all the genes from these 3 modules and also constructed the PPI network (Fig. 3E).

### Building the prognostic RBPs-based signature

**Fig. 4:**
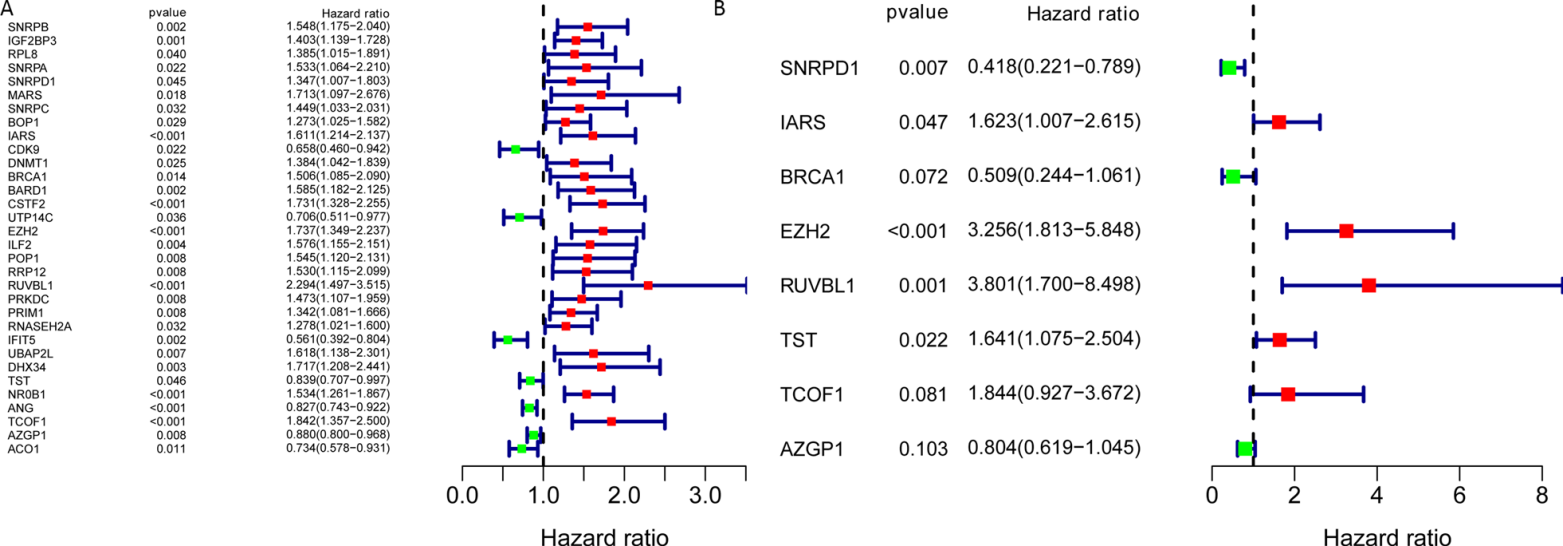
Univariate and multivariate COX analysis to selected independent prognosis RNA binding proteins of HCC patients. **A** Univariate; **B** multivariate

To screen the prognostic RBPs, differently expressed RBPs were conducted in univariate COX analysis. Then, 32 RBPs of great significance in univariate COX analysis were included in multivariate COX analysis. Finally, 8 differently expressed RBPs (*SNRPD1, IARS, BRCA1, EZH2, RUVBL1, TST, TCOF1,* and *AZGP1*) were selected as independent prognosis factors of HCC patients in the training set Fig. 4. Thus, the formula for our model was: Risk Score = (0.484 × expression _IARS_) + (0.180 × expression_EZH2_) + (1.335 × expression_RUVBL1_) + (0.495 × expression_TST_) + (0.612 × expression_TCOF1_) − (0.872 × expression_SNRPD1_) − (0.676 × expression_BRCA1_) − (0.218 × expression_AZGP1_). In addition, in our study cohort, the risk score of individual patients was estimated. Using the median risk score value as a cut-off the cohorts were then divided into high- and low-risk groups in training set and testing set.

### Survival results and multivariate examination

**Fig. 5:**
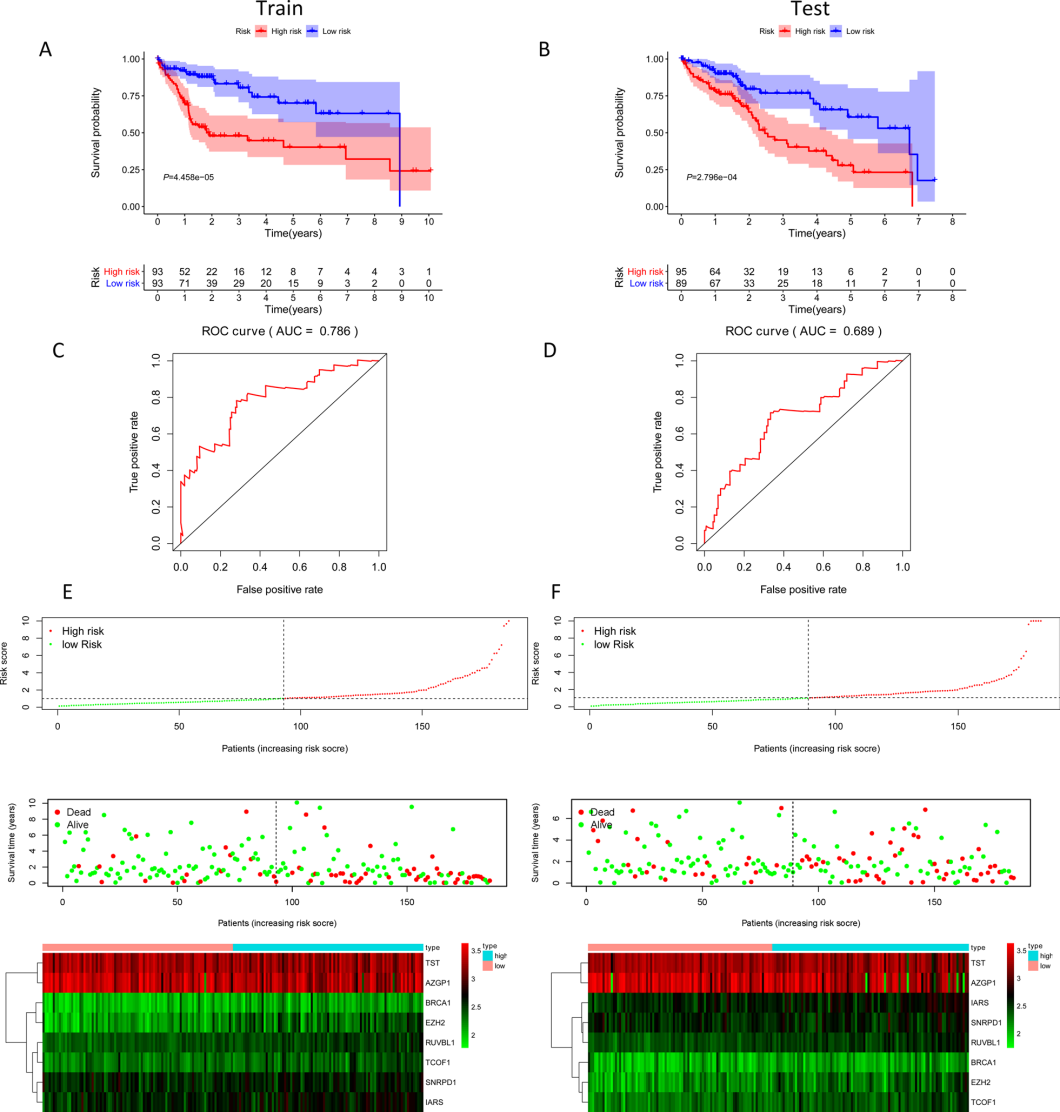
Survival results and multivariate examination in training set and testing set. **A** Survival results in training set; **B** survival results in testing set; **C** ROC result in training set; **D** ROC result in testing set; **E** risk survival status plot in training set; **F** risk survival status plot in testing set

**Fig. 6:**
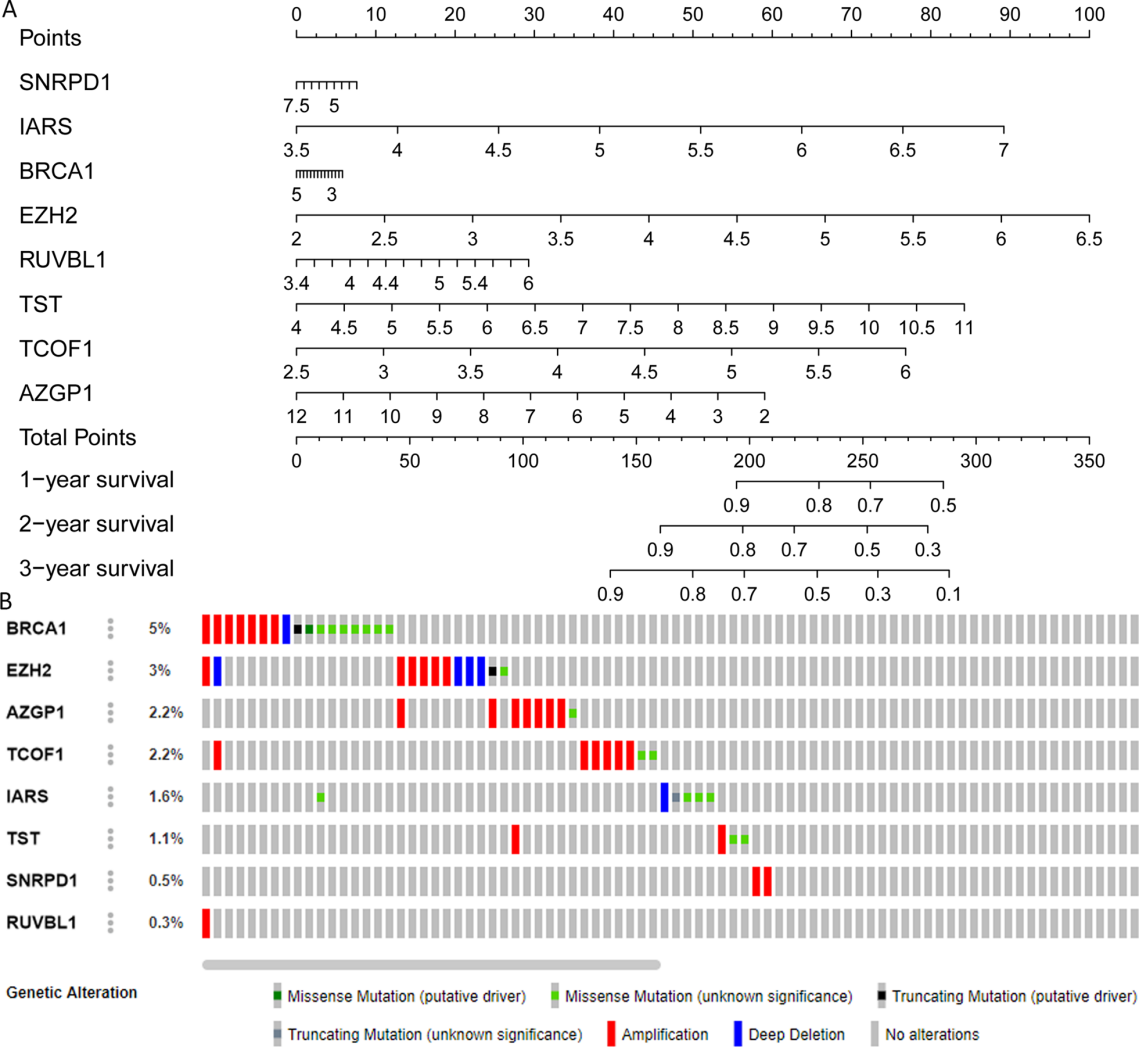
A nomogram and mutation and copy-number alteration (CNA) analyses of the 8 RBPs*.* **A** Nomogram; **B** cBioPortal results

The Kaplan–Meier curves of the two cohorts can detect the predictive value of the signature based on 8-RBPs in the OS. Patients with high risk have a bad survival than the low-risk group both in training cohort (*P* = 4.458e − 05, Fig. 5A) and testing cohort (*P* = 2.796e − 04, Fig. 5B). We also used the ROC curves to investigate whether the expression pattern of survival-related RBPs could provide an early prediction for the occurrence of HCC. Here, we found an AUC of 0.786 in training set and 0.689 in testing set, meaning that the sensitivity and specificity of this prognostic model are moderate Fig. 5C, D. At the same time, we also established patient’s risk survival status plot, and as the patient's risk score increases, the number of dead patients also increases Fig. 5E, F. Next, in order to establish a prognostic model based on 8-RBPs, univariate and multivariate COX analysis was used to determine risk factors. Ultimately, the 8-RBPs based signature were convinced as independent prognosis factors of OS Table 1. Finally, combined with clinical pathology and prognostic models, a nomogram was constructed. Combining our prognostic model with clinical pathology can improve the predictive sensitivity and specificity of OS predictions at 1, 2, and 3 years, and bring some net benefits that may be useful for clinical management Fig. 6A. To test the robustness of the signature constructed from the TCGA cohort, the patients from the ICGC cohort were also categorized into high- or low-risk groups by the median value calculated with the same formula as that from the TCGA cohort. Similarly, patients in the high-risk group were more likely to encounter death earlier and the AUC of the 10-gene signature was 0.705. We also established patient's risk survival status plot, and as the patient's risk score increases, the number of dead patients also increases Additional file 1: Figure S1.

**Table 1:** The univariate and multivariate COX analysis results to identify risk factors

	Univariate		Multivariate	
Item	HR (HR.95L–HR.95H)	*P* value	HR (HR.95L–HR.95H)	*P* value
Training set				
Age	1.011 (0.991−1.031)	0.284	0.998 (0.978−1.019)	0.865
Gender	0.908 (0.529−1.558)	0.725	2.049 (1.061−3.955)	0.033
Grade	1.098 (0.769−1.566)	0.608	1.177 (0.793−1.745)	0.419
Stage	1.536 (1.117−2.112)	0.008	0.247 (0.025−2.401)	0.228
T	1.471 (1.094−1.978)	0.011	4.252 (0.469−38.512)	0.198
M	1.183 (0.871−1.607)	0.283	0.931 (0.634−1.366)	0.714
N	1.026 (0.746−1.411)	0.875	0.883 (0.589−1.324)	0.547
riskScore	1.438 (1.280−1.617)	< 0.001	1.234 (1.057−1.441)	0.008
Testing set				
Age	1.012 (0.988−1.036)	0.338	0.980 (0.955−1.004)	0.106
Gender	0.652 (0.370−1.150)	0.139	1.360 (0.717−2.578)	0.347
Grade	1.159 (0.792−1.695)	0.447	1.256 (0.784−2.012)	0.343
Stage	1.724 (1.307−2.272)	< 0.001	0.666 (0.230−1.924)	0.452
T	1.749 (1.340−2.282)	< 0.001	1.414 (0.512−3.906)	0.504
M	1.159 (0.852−1.577)	0.346	1.738 (1.097−2.756)	0.019
N	1.098 (0.809−1.492)	0.548	0.799 (0.522−1.222)	0.300
riskScore	1.189 (1.084−1.304)	< 0.001	1.180 (1.058−1.316)	0.003

### Online database analysis

**Fig. 7:**
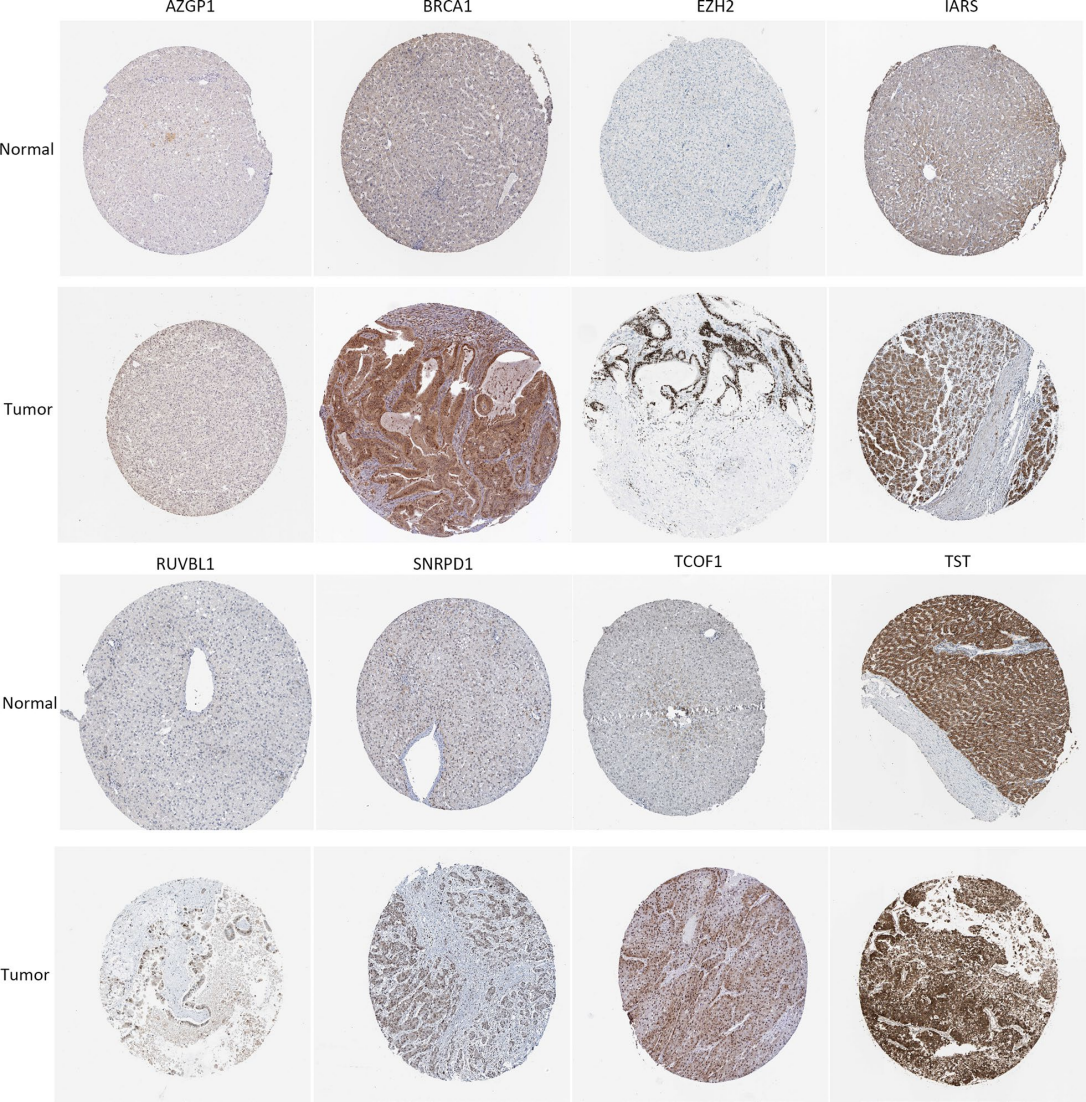
The immunohistochemistry results from the HPA database of *SNRPD1, IARS, BRCA1, EZH2, RUVBL1, TST, TCOF1,* and *AZGP1*

**Fig. 8:**
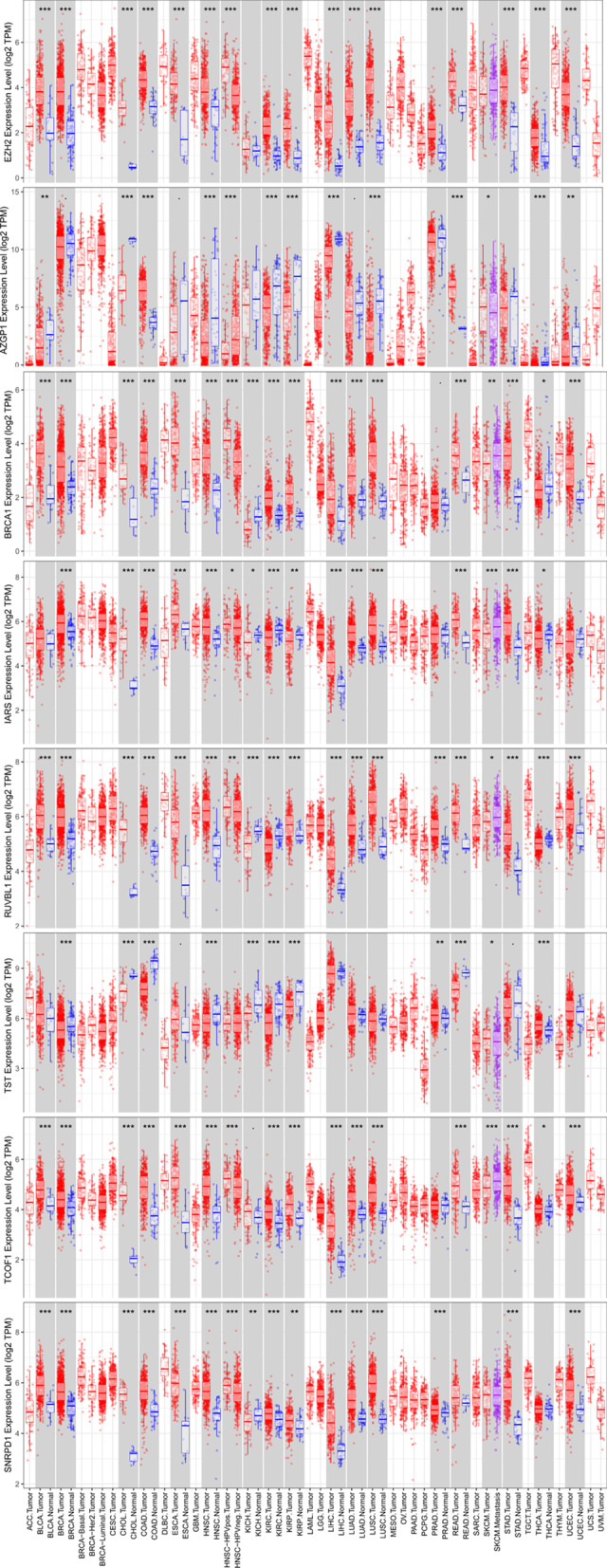
The differential expression between tumor and adjacent normal tissues for *SNRPD1, IARS, BRCA1, EZH2, RUVBL1, TST, TCOF1* and *AZGP1* across all TCGA tumors

**Fig. 9:**
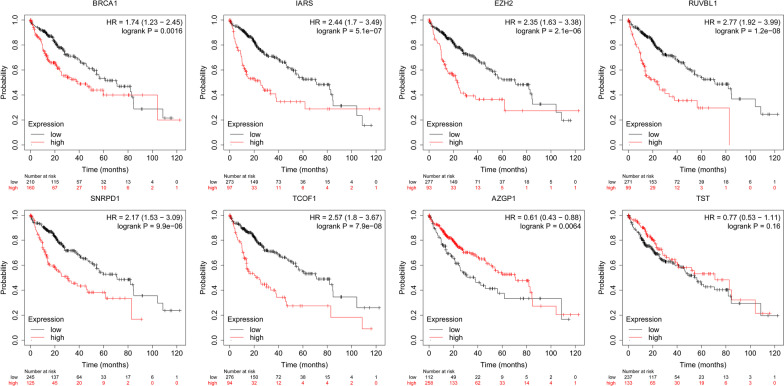
Kaplan–Meier plotter to analyze the prognostic relevance of *SNRPD1, IARS, BRCA1, EZH2, RUVBL1, TST, TCOF1,* and *AZGP1*

Mutation and copy-number alteration (CNA) of these 8 RPPs conducted by the cBioPortal online tool. The results demonstrated that these genes were altered in 50 samples out of 357 HCC patients (14%) and the amplification of *BRCA1* was the largest frequent copy-number alteration Fig. 6B. In order to further analysis the expression of these 8 RBPs, immunohistochemistry results from the HPA database to illustrate that *SNRPD1, IARS, BRCA1, EZH2, RUVBL1, TST, TCOF1,* and *AZGP1* were significantly increased in tumor tissues Fig. 7. Meanwhile, we also used TIMER database to study the differential expression between tumor and adjacent normal tissues for these 8 RBPs across all TCGA tumors Fig. 8. We finally used Kaplan–Meier plotter to analyze the prognostic relevance of the these 8 RBPs, the results indicated that the high expression of *SNRPD1, IARS, BRCA1, EZH2, RUVBL1,* and *TCOF1* was related to a poor prognostic, while the high expression of *AGZP1* was related to a good prognostic and the expression of *TST* was meaningless to HCC patient’s overall survival Fig. 9.

## Discussion

Alterations in post-transcriptional events are a critical step in tumor initiation and progression, while RBP-mediated control has not yet well been developed in cancers. RBPs has a wide range of functions, including regulation of mRNA’s stability/splicing/editing/translation/export/positioning/polyadenylation and miRNA biogenesis, which ultimately affect the expression of individual gene and serve as the key molecular connections in cancer [12]. Therefore, understanding the basic mechanisms of RBPs regulation may provide useful insights for the development of effective cancer treatments. In this study, we determined a novel and effective RBPs-based signature prognostic using online data set, and our signature may act for the RBPs status of HCC patients and provide potential biomarkers for clinical therapeutic intervention.

In our study, a comprehensive analysis of RBPs as well as downloaded clinical data from HCC-TCGA has been studied. We first identified 208 upregulated and 122 downregulated RBPs in HCC patients and explored the functions of these RBPs. Gene ontology function were mainly enriched in post-transcriptional events and KEGG were mainly enriched in spliceosome, mRNA surveillance pathway, RNA transport, RNA degradation, Influenza A, Hepatitis C, RIG-I-like receptor signaling pathway and Herpes simplex virus 1 infection. The retinoic acid-inducible gene I (RIG-I)-like receptors (RLRs) belong to the cytosolic host RNA helicases family that identify distinct nonself RNA signatures and trigger innate immune responses against some RNA viruses by signaling via the required adaptor protein mitochondrial antiviral signaling (MAVS) [13]. Innate immune activation and signaling through the RLRs pathway relied on viral replication, where the host response can obviously limit replication in target cells [14]. It’s well known that virus infection palys an indispensable role in HCC occurrence and development. Our results found the downregulated RBPs mainly enriched in viruses-related signaling pathway and may provide novel insight into further researches in future.

We next identified 8 RBPs (*SNRPD1, IARS, BRCA1, EZH2, RUVBL1, TST, TCOF1,* and *AZGP1*) which were selected as independent prognosis factors of HCC patients. Small nuclear ribonucleoprotein D1 polypeptide (*SNRPD1*), which encodes a small nuclear ribonucleoprotein is a part of the SNRNP core protein family. The mutations *SNRPD1* antigenic targets are related to autologous T cell responses in the melanoma model [15]. SiRNA-mediated depletion of *SNRPD1* leads to a significant reduction in cell viability in breast cancer, lung cancer, and leads to autophagy by mTOR pathway [16]. Kopajtich et al. [17] found that isoleucine-tRNA synthetase (*IARS*) missense mutations can cause hereditary weak calf syndrome and thus lead to infantile hepatopathy. It has been also confirmed in oral squamous cell carcinoma tissues that *IARS* levels involved in aminoacyl tRNA biosynthesis were elevated [18]. *BRCA1* DNA repair associated (*BRCA1*) is a tumor suppressor which maintaining genomic stability and associates with RNA polymerase II. A recent study indicated that PARP inhibitor (olaparib), could increase the cytotoxicity in HCC cells with a lower *BRCA1* expression [19]. The enhancer of zeste homolog 2 (*EZH2*) and the catalytic component of polycomb inhibition complex 2 (*PRC2*) are associated with the expression of homologous genes (Hox) and early stages of inactivating X chromosome [20]. HBV HBx regulatory protein and lncRNA DLEU2 co-recruitment on the cccDNA replaces *EZH2* from the viral chromatin to promote transcription and viral replication [21]. RuvB like AAA ATPase 1 (*RUVBL1*) is a protein that has both DNA-dependent ATPase and DNA helicase activities. It regulates insulin signaling via the Akt/mTOR pathway in vivo and in normal liver cells and HCC cells in vitro [22]. Thiosulfate sulfurtransferase (*TST*), also known as Rhodanese, interact with 5S ribosomal RNA and promote its into mitochondria. *TST* like domain-containing 1 (*TSTD1*) might play a role in sulfide-based signaling and overexpression in colon cancer [23]. *TST*’s functions also include modification of iron-sulfur clusters, sulfur metabolism, and the reduction of antioxidant glutathione and thioredoxin [24]. Treacle ribosome biogenesis factor 1 (*TCOF1*) participates in ribosomal DNA gene transcription and maintains genomic integrity after DNA damage [25]. Heterozygous mutations of *TCOF1* are related to craniofacial disorder and compromise nucleolar homeostasis, thus activate the tumor-suppressor protein [26]. Study found that alpha-2-glycoprotein 1 (*AZGP1*) inhibited HCC cell invasion and migration by the regulation of the *PTEN/Akt* and *CD44s* pathways [27]. The absence of *AZGP1* may trigger epithelial-mesenchymal transition (EMT) induced by TGFβ1-ERK2 signaling [28]. In total, the RBPs identified by our study may plays a critical role in the HCC occurrence and development.

Meanwhile, we also using online database to predict the potential function of these 8 RBPs including mutation and copy-number alteration; immunohistochemistry expression and pan-cancer expression. Though post-transcriptional plays a critical role in HCC, RBPs that affected genes still are unclear. In this study, we tried to integrates some RBPs biomarkers to assess the prognosis of treatment effects. This model can promote the determination of novel biomarkers and precise medical targets of diseases in HCC. Meanwhile, the model can also help with prognosis prediction, diagnosis and strategies of patients with distinct epigenetic subtypes of HCC. The signatures in our study might offer potential useful biomarkers for cancer treatment and prediction therapy response. However, our signatures have to prove in further independent cohorts and predictive RBPs functional by experiments. Meanwhile, our research has limitations. Firstly, our outcomes have not been validated in clinical samples. Secondly, our results do not provide accurate clinical data due to the relatively small number of patients. Finally, due to our RBPs were got from Gerstberger et al. study, some information may be inaccurate, such as *BRCA1* may not belong to RBPs, so it should be caution when clarifying our research. Although our research wishes to explored the probability of establishing a prognostic prediction model, it is still in its infancy and needs improvement.

## Conclusion

In conclusion, from the TCGA database and other bioinformatics ways, we have found prognostic RBPs as well as built a prognostic prediction model for HCC patients. The model might assist us identify novel biomarkers and help to predict prognosis of disease, clinical diagnosis and management.

## Supplementary Information

**Additional file 1**. Figure S1. Survival results and multivariate examination in ICGC cohort. A: Survival results in ICGC; B: ROC results in ICGC; C: Risk survival status plot in ICGC.

**Additional file 2**. Table S1: The detailed information of 1,542 RNA binding proteins. Table S2: The detailed information of 330 differently expressed RBPs (208 upregulated and 122 downregulated). Table S3: The detailed information of function of the identified upregulated and downregulated RNA binding proteins, respectively.

## Data Availability

The scripts (Perl and R) used to calculate read counts mapped to Refseq-defined exons and introns are available at https://github.com/tianjiagene/GneneBlockReadCount. The two public RNA-seq data were from TCGA database and ICGC database.
