# Supplementary figures and images for: Integrated analysis of the functions and prognostic values of RNA binding proteins in hepatocellular carcinoma
Source: BMC Gastroenterol. 2021 Jun 15;21:265. doi: 10.1186/s12876-021-01843-0 (PMC8204501; doi:10.1186/s12876-021-01843-0)

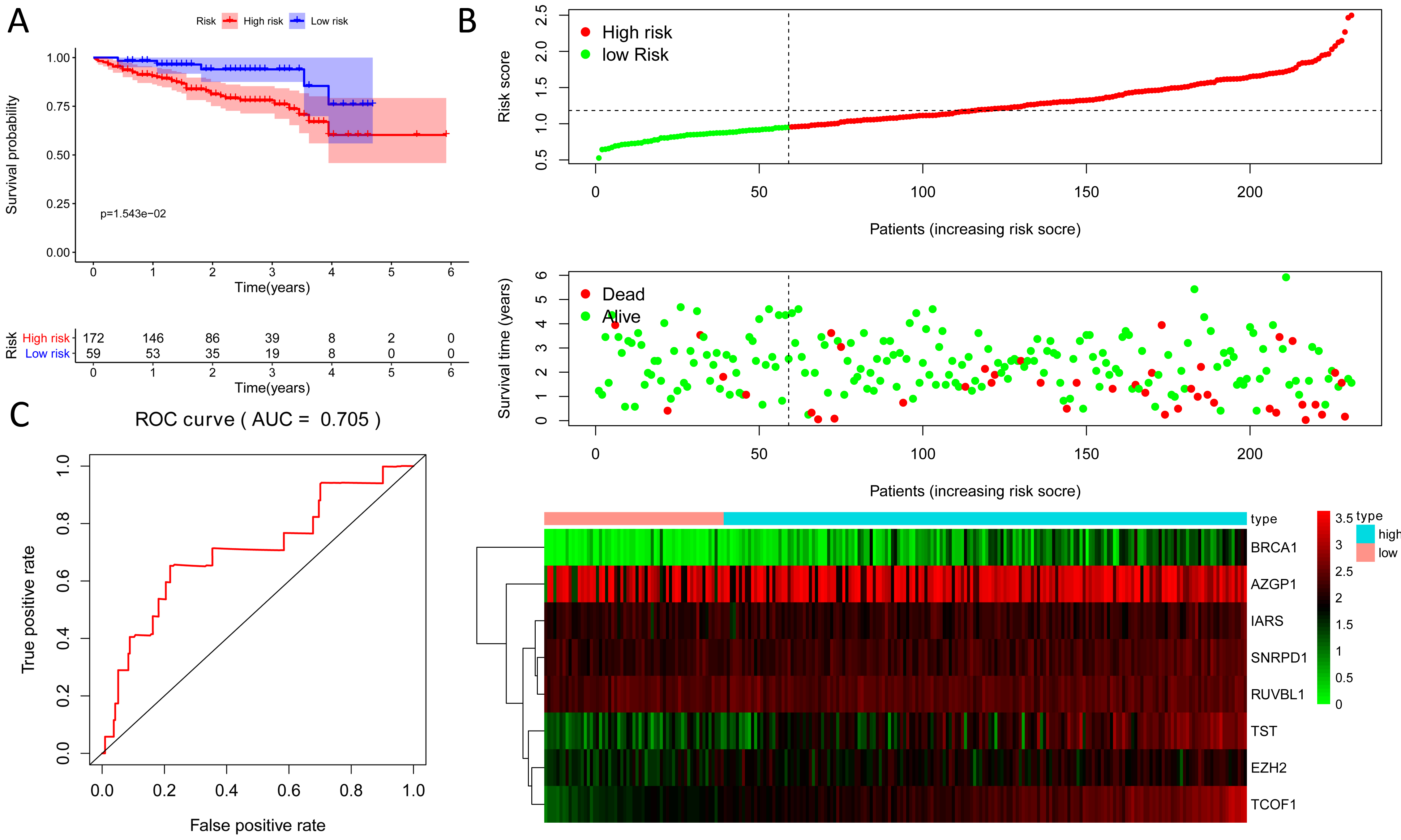

Supplement: Supplementary file 1 — Additional file 1. Figure S1. Survival results and multivariate examination in ICGC cohort. A: Survival results in ICGC; B: ROC results in ICGC; C: Risk survival status plot in ICGC. [file 12876_2021_1843_MOESM1_ESM.jpg]
